# Supplementary material for: Signals interpreted as archaic introgression appear to be driven primarily by faster evolution in Africa
Source: R Soc Open Sci. 2020 Jul 1;7(7):191900. doi: 10.1098/rsos.191900 (PMC7428223; doi:10.1098/rsos.191900)
Supplement: example C++ code used to parse and analyse the vcf files [file rsos191900supp1.rtf]

// hangover headers, don't need all but was lazy and did not delete unneeded#include <iostream>#include <stdlib.h>#include <fstream>#include <string.h>#include <stdio.h>#include <math.h>#include <time.h>using namespace std;char LoadChimp(ifstream& in3, long location, char rf);void LoadPops();void ABBA_IND(char Nea, char Cp, char at, char rf, int geno[2504], float frq[26][3]);char infer(int b[4]);char line[1000000];int pops[2504][2]{};int N_ind[26]{};float P_val[3][3][2][2]{};float ABBAtype[26][26][6][2]{};int main(){	// read population origin data for all 2504 individuals, stored as pops[individual][0 = pop, 1 = region]	LoadPops();	// take the chromosome to analyse as a string and convert to a number	char temp[10000];	int chrom;	cout << "chromosome to analyse ";	cin >> temp;	if (temp[1] == '\0') chrom = temp[0] - 48;	else chrom = 10 * (temp[0] - 48) + temp[1] - 48;	// open file of Neanderthal bases, stored as location, counts for A, C, G and T	char infile1[200] = "B:\\Neand_CHR";	strcat_s(infile1, temp);	strcat_s(infile1, ".txt");	ifstream in1(infile1);	if (in1) cout << infile1 << " is open\n";	else cout << infile1 << "is not open\n";	// open vcf file of 1000 genome data and remove header information	// note, I have changed the name of downloaded, decompressed vcf file to 'ALL.CHR[chrom].vcf'	char infile2[200] = "B:\\All.CHR";	strcat_s(infile2, temp);	strcat_s(infile2, ".vcf");	ifstream in2(infile2);	if (in2) cout << infile2 << " is open\n";	else cout << infile2 << " is not open\n";	for (int i = 0; i < 254; i++) in2.getline(line, 1000000);	// open file of chimpanzee bases	char infile3[200] = "B:\\chr";	strcat_s(infile3, temp);	strcat_s(infile3, "CH.txt");	ifstream in3(infile3);	if (in3) cout << infile3 << " is open\n";	else cout << infile3 << " is not open\n";	// open file for output	char ofile1[200] = "F:\\Conditioned_D_CHR";	strcat_s(ofile1, temp);	strcat_s(ofile1, ".txt");	ofstream out1(ofile1);	if (out1) cout << ofile1 << "  open\n";	else cout << ofile1 << " is not open\n";	long locHUM = 0, locCMP = 0, locNEA = 0; // variables for locations of each site	int lcn = 0, prev = 0;	char ref2, ref3, NEA, CMP;	long long psn = 0;	// work through, parsing the human vcf file	while (!in2.eof()) {		char alt[1000], ref[1000];		in2 >> temp >> locHUM >> temp >> ref >> alt >> temp >> temp >> temp;		// generate location in whole maegabases and test for an increase to display progress		lcn = locHUM / 1000000;		if (lcn > prev) cout << lcn << "\n"; // output a megabase count		prev = lcn;		bool SNP = false;		int g = 0;		// test whether variant type is a SNP		while (temp[g] != '\0') {			if (temp[g] == 'V' & temp[g + 1] == 'T' & temp[g + 2] == '=' & temp[g + 3] == 'S' & temp[g + 4] == 'N') SNP = true;			g++;		}		// if reference and alternate alleles are single bases		if (alt[1] == '\0' & SNP & ref[1] == '\0') {			// extract Denisovan, Neanderthal and chimpanzee bases 			NEA = '-', CMP = '-';			int base[4]{};			while (!in1.eof() & locNEA < locHUM) {				in1 >> locNEA >> ref2 >> base[0] >> base[1] >> base[2] >> base[3];				if (locNEA >= locHUM) break;			}			if (locNEA == locHUM) NEA = infer(base);			while (!in3.eof() & locCMP < locHUM) {				in3 >> locCMP >> ref3 >> CMP;				if (locCMP != locHUM) CMP = '-';				if (locCMP > locHUM) break;			}			if (CMP != '-' & NEA != '-') { // if all bases called				in2 >> temp;				in2.getline(line, 100000);  // read genotype fields into ‘line’				float freq[26][3]{};				int gen[2504]{};				// store genotypes and count alleles in each population				for (int g = 0; g < 2504; g++) {					int a1 = line[g * 4 + 1] - 48;					int a2 = line[g * 4 + 3] - 48;					gen[g] = a1 + a2; // 0 = homozygote Ref, 1 = heterozygote, 2 = homozygote alternate					freq[pops[g][0]][a1 + a2]++;				}				for (int pop = 0; pop < 26; pop++) { // frequencies of each genotype in each pop					for (int i = 0; i < 3; i++) freq[pop][i] = freq[pop][i] / N_ind[i];				}				if ((NEA == alt[0] & CMP == ref[0]) | (NEA == ref[0] & CMP == alt[0])) {					ABBA_IND(NEA, CMP, alt[0], ref[0], gen, freq); // D calculations				}			}			else in2.getline(line, 1000000);		}		else in2.getline(line, 1000000);	}	// output to file in 9 column blocks for compactness	for (int p1 = 0; p1 < 26; p1++) {		for (int p2 = p1; p2 < 26; p2++) {			out1 << p1 << "\t" << p2;			for (int i = 0; i < 6; i++) {				out1 << "\t" << ABBAtype[p1][p2][i][0] << "\t" << ABBAtype[p1][p2][i][1];			}			out1 << "\n";		}	}	out1.close();}// main calculations for Dvoid ABBA_IND(char Nea, char Cp, char at, char rf, int geno[2504], float frq[26][3]){	long c = 0;	int N = -1;	if (Nea == rf) N = 0;	if (Nea == at) N = 1;	if (N > -1) {		// compare each pop against every other pop		for (int pop1 = 0; pop1 < 26; pop1++) {			for (int pop2 = pop1; pop2 < 26; pop2++) {				// rotate all 9 possible 2-locus genotypes coded 0 = Ref/Ref, 1 = Ref/Alt, 2 = Alt/Alt				for (int i = 0; i < 3; i++) {					for (int j = 0; j < 3; j++) {						int typ = 0; // no conditioning						ABBAtype[pop1][pop2][typ][0] += frq[pop1][i] * frq[pop2][j] * P_val[i][j][N][0];						ABBAtype[pop1][pop2][typ][1] += frq[pop1][i] * frq[pop2][j] * P_val[i][j][N][1];						if (j != 1) { // second locus is homozygous (i.e. not heterozygous)							typ = 1;							ABBAtype[pop1][pop2][typ][0] += frq[pop1][i] * frq[pop2][j] * P_val[i][j][N][0];							ABBAtype[pop1][pop2][typ][1] += frq[pop1][i] * frq[pop2][j] * P_val[i][j][N][1];						}						if (j == 1) { // second locus is heterozygous							typ = 2;							ABBAtype[pop1][pop2][typ][0] += frq[pop1][i] * frq[pop2][j] * P_val[i][j][N][0];							ABBAtype[pop1][pop2][typ][1] += frq[pop1][i] * frq[pop2][j] * P_val[i][j][N][1];						}						if (i != 1) { // first locus is homozygous (not heterozygous)							typ = 3;							ABBAtype[pop1][pop2][typ][0] += frq[pop1][i] * frq[pop2][j] * P_val[i][j][N][0];							ABBAtype[pop1][pop2][typ][1] += frq[pop1][i] * frq[pop2][j] * P_val[i][j][N][1];						}						if (i == 1) { // first locus is heterozygous							typ = 4;							ABBAtype[pop1][pop2][typ][0] += frq[pop1][i] * frq[pop2][j] * P_val[i][j][N][0];							ABBAtype[pop1][pop2][typ][1] += frq[pop1][i] * frq[pop2][j] * P_val[i][j][N][1];						}						if (i != 1 & j != 1) { // both loci are homozygous							typ = 5;							ABBAtype[pop1][pop2][typ][0] += frq[pop1][i] * frq[pop2][j] * P_val[i][j][N][0];							ABBAtype[pop1][pop2][typ][1] += frq[pop1][i] * frq[pop2][j] * P_val[i][j][N][1];						}					}				}			}		}	}}char infer(int b[4]) // if base counts are between 10 and 200 and >80% are of one base return that base{	float tot = b[0] + b[1] + b[2] + b[3];	if (tot > 9 & tot < 250) {		if (float(b[0]) / tot > 0.8) return 'A';		else if (float(b[1]) / tot > 0.8) return 'C';		else if (float(b[2]) / tot > 0.8) return 'G';		else if (float(b[3]) / tot > 0.8) return 'T';	}	return '-';}char LoadChimp(ifstream& in3, long location, char rf){	char cmp = '-';	char ref3 = '-';	long location3 = 0;	while (!in3.eof() & location3 < location) {		long long psn1 = in3.tellg();		in3 >> location3;		if (location3 < location) in3.getline(line, 10000);		if (location3 > location) in3.seekg(psn1, ios::beg);	}	if (location == location3) {		in3 >> ref3 >> cmp;		if (ref3 != rf) cout << "**cmp mismatch**";	}	return cmp;}void LoadPops() // read in population and region codes for each of 2504 individuals{	char infile1[200] = "B:\\inpops.txt";	ifstream in1(infile1);	in1.getline(line, 1000);	for (int i = 0; i < 2504; i++) {		in1 >> line;		in1 >> pops[i][0];		in1 >> pops[i][1];		pops[i][0]--; // population codes start at 1 so decrement for use as array indices		pops[i][1]--;		N_ind[pops[i][0]] ++; // counts alleles scored in each population		in1.getline(line, 200);	}	in1.close();	// set precalculated probabilities for ABBA and BABA: P_val[geno1][geno2][Nea base][ABBA / BABA]	P_val[0][0][0][0] = 0, P_val[0][1][0][0] = 0, P_val[0][2][0][0] = 0;	P_val[0][0][0][1] = 0, P_val[0][1][0][1] = 0.5, P_val[0][2][0][1] = 1;	P_val[0][0][1][0] = 0, P_val[0][1][1][0] = 0.5, P_val[0][2][1][0] = 1;	P_val[0][0][1][1] = 0, P_val[0][1][1][1] = 0, P_val[0][2][1][1] = 0;	P_val[1][0][0][0] = 0.5, P_val[1][1][0][0] = 0.25, P_val[1][2][0][0] = 0;	P_val[1][0][0][1] = 0, P_val[1][1][0][1] = 0.25, P_val[1][2][0][1] = 0.5;	P_val[1][0][1][0] = 0, P_val[1][1][1][0] = 0.25, P_val[1][2][1][0] = 0.5;	P_val[1][0][1][1] = 0.5, P_val[1][1][1][1] = 0.25, P_val[1][2][1][1] = 0;	P_val[2][0][0][0] = 1, P_val[2][1][0][0] = 0.5, P_val[2][2][0][0] = 0;	P_val[2][0][0][1] = 0, P_val[2][1][0][1] = 0, P_val[2][2][0][1] = 0;	P_val[2][0][1][0] = 0, P_val[2][1][1][0] = 0, P_val[2][2][1][0] = 0;	P_val[2][0][1][1] = 1, P_val[2][1][1][1] = 0.5, P_val[2][2][1][1] = 0;}
